# Evaluation of Medical Care for Diabetic and Hypertensive Patients in Primary Care in Mexico: Observational Retrospective Study

**DOI:** 10.1155/2021/7365075

**Published:** 2021-08-14

**Authors:** Agustin Lara-Esqueda, Sergio A. Zaizar-Fregoso, Violeta M. Madrigal-Perez, Mario Ramirez-Flores, Daniel A. Montes-Galindo, Margarita L. Martinez-Fierro, Iram P. Rodriguez-Sanchez, José Guzman-Esquivel, Carmen Meza-Robles, Gabriel Ceja-Espiritu, Pablo A. Kuri-Morales, Josuel Delgado-Enciso, Armando Barriguete-Melendez, Hector R. Galvan-Salazar, Carlos E. Barajas-Saucedo, Elvin Guillermo-Espinosa, Agustin D. Lara-Basulto, Jesus F. Gonzalez-Roldan, Ivan Delgado-Enciso

**Affiliations:** ^1^Department of Research, Cancerology State Institute, Colima State Health Services, Colima 28085, Mexico; ^2^Department of Molecular Medicine, School of Medicine, University of Colima, Colima 28040, Mexico; ^3^Molecular Medicine Laboratory, Academic Unit of Human Medicine and Health Sciences, Zacatecas Autonomous University, Zacatecas 98160, Mexico; ^4^Molecular and Structural Physiology Laboratory, School of Biological Sciences, Autonomous University of Nuevo León, Monterrey, Nuevo León 64460, Mexico; ^5^Department of Research, Mexican Social Security Institute, Villa de Alvarez, Colima 28983, Mexico; ^6^School of Medicine, Universidad Nacional Autonoma de Mexico, Ciudad de Mexico, 04510, Mexico; ^7^Fundación para la Etica, Education e Investigación del Cáncer del Instituto Estatal de Cancerologia de Colima, Colima 28085, Mexico; ^8^Universidad Anahuac Ciudad de Mexico, Mexico; ^9^Colima State Health Services, Colima 28085, Mexico; ^10^Subsecretaria de Prevención y Promoción de la Salud, Secretaria de Salud de Mexico, Ciudad de Mexico, 06600, Mexico

## Abstract

**Introduction:**

The present study evaluated the quality of medical care for patients diagnosed with diabetes mellitus (DM), hypertension (HBP), and both pathologies (DM+HBP) within a public health system in Mexico.

**Methods:**

45,498 patients were included from 2012 to 2015. All information was taken from the electronic medical record database. Each patient record was compared against the standard to test the quality of medical care.

**Results:**

Glycemia with hypertension goals reached 29.6% in DM+HBP, 48.6% in DM, and 53.2% in HBP. The goals of serum lipids were reached by 3% in DM+HBP, 5% in DM, and 0.2% in HBP. Glycemia, hypertension, and LDL cholesterol reached 0.04%. 15% of patients had an undiagnosed disease. Clinical follow-up examinations reached 20% for foot examination and clinical eye examination. Specialty referrals reached 1% in angiology or cardiology.

**Conclusion:**

Goals for glycemic and hypertension reached 50% in the overall population, while serum lipids, clinical follow-up examinations, and referral to a specialist were deficient. Patients who had both diseases had more consultations, better control for hypertension and lipids, but inferior glycemic control. Overall, quality care for DM and/or HBP has not been met according to the standards.

## 1. Introduction

Diabetes mellitus (DM) is characterized by hyperglycemia resulting from defects in insulin secretion, insulin action, or both. Chronic hyperglycemia is associated with long-term damage, dysfunction, and organ failure, especially in the eyes, kidneys, nerves, heart, and blood vessels [1].

DM is a worldwide health problem where Mexico holds sixth place in prevalence globally, given that 15% of the adult population above 20 years of age had been diagnosed [2]. A national survey of the Mexican population showed that 54.5% of DM patients had visual acuity impairment, 9.9% had vision loss, 9.14% presented with foot ulcers, and 5.5% had undergone amputation of an extremity [3].

DM is also the leading cause of premature death within the Mexican population [4], holding the highest diabetes-specific mortality rate in Latin America, where 50% of the patients with DM died from cardiovascular events [5]. Moreover, the presence of DM increases the risk of developing hypertension (HBP) [6].

HBP prevalence within DM patients varies from 30% to 60% [7] and causes up to a 37-fold increase in mortality [8]. Comparing DM patients against non-DM patients, controlled hypertension has been demonstrated to improve risk [9].

Franklin et al. found that HBP was not diagnosed in 29% of DM patients, which had a higher risk (1.5 times) for presenting a cardiovascular event [10]. Consequently, HBP becomes another worldwide public health problem. In Mexico, 31% of the adult population above 20 years of age presents with HBP [11]. 45% of patients diagnosed with HBP died from a cardiac ischemic event, and 51% died from a cerebral ischemic event [12].

Long and Dagogo-Jack reported higher rates of cardiovascular death, myocardial infarction, angina pectoris, amputation, and stroke when patients with DM+HBP were compared against DM-only patients [13].

HBP and DM's economic impact is an enormous burden on society, with an estimated annual cost of $174 billion for DM care and $76.6 billion for HBP-related problems [13]. Both have a highly negative impact on the quality of life of patients, the family nucleus, and the country's economy [14]. Opportune diagnosis and treatment are relevant points for reducing associated complications and the probability of premature death [15].

Patients with DM+HBP have a higher risk of complications. Therefore, targeting multiple risk factors is essential to preventing and slowing the progression of these complications. Optimization of glycemic, lipid, and hypertension control has been demonstrated to improve patient outcomes [13].

The present study took DM+HBP patients as the pivot point to evaluate the quality of medical care given to them by comparing them against DM-only and HBP-only patients. The concept of quality of medical care is a problematic notion of defining. It is described herein as the compliance with actions and goals stated in the Mexican Clinical Practice Guidelines [16, 17] and the Official Mexican Standards [18, 19], which are based on the international guidelines of the American Diabetes Association (ADA) for diabetes [20] and the Eighth Joint National Committee (JNC 8) for hypertension [21].

## 2. Methods

### 2.1. Study Population

Data were obtained from the electronic health record (EHR) of Colima, known as SAECCOL (Spanish acronym), from January 2012 to December 2015. The SAECCOL was certified in the NOM-024-SSA3-2012, and its implementation and benefits have been previously described [22].

The present study was approved by the Research Ethics Committee of the Colima State Cancerology Institute, Colima State Health Services, Colima, Mexico. According to the World Medical Association Declaration of Helsinki and the General Guidelines for Health Research, it was carried out. The anonymity of the patients and healthcare personnel was guaranteed.

This study's inclusion criteria were to be diagnosed with diabetes and/or hypertension before December 2015 and stated in the EHR. No previous records before diagnosis were included. Therefore, patients without any of the two diseases were excluded.

Second, patients must be above 18 years old and have at least one record in the EHR. The study included 45,498 patients diagnosed with DM and/or HBP in the EHR within the study period. The population was divided into three categories: (a) diabetes mellitus only (DM), (b) hypertension only (HBP), and (c) diabetes mellitus plus hypertension (DM+HBP). All three categories were taken as stated in the EHR by a single categorical value, 0 for not having the diagnosis and 1 for diagnosis.

### 2.2. Healthcare Goals

The definition of the goals related to disease control and the procedures undergone by the patients were taken from the Mexican Clinical Practice Guidelines [16, 17] and the Official Mexican Standards [18, 19], which are based on international guidelines of the ADA for diabetes [20] and the JNC 8 for hypertension [21] (see Table 1).

Although international guidelines establish Hb1Ac as one of the goals, this method has not been adopted as a standard routine inside the Mexican public health system. Glucose was measured as preprandial with eight hours of fasting before the blood sample, and the target value was agreed upon lower than 126 mg/dl after guideline consulting. Postprandial glucose was measured two hours after 75 mg of oral glucose, and the target value was agreed upon lower than 200 mg/dl after guideline consulting. Clinical foot examination was identified as the performance of at least one clinical exam concerning deep and superficial lower limb sensitivity. Clinical eye examination was regarded as ophthalmoscopy performed in the primary care setting. Angiology, cardiology, internal medicine, and ophthalmology referrals were registered when the primary care physician referred the patient to the medical specialist, whether or not the patient went to that consultation.

### 2.3. Data Processing

The anonymity of the patients and healthcare personnel was guaranteed. Data cleansing was carried out for each variable, eliminating extreme values that could be part of an erroneous capture. Missing values were substituted with 0 or any representative value that meant no action was performed for that measurement. The Mexican norms state that if an action is not registered in the clinical case record, it is treated as if it did not occur [23].

Finally, variables were grouped for each patient, synthesizing four years of records into a single register per patient, turning the analysis into a cross-sectional study. This measure had to be made given the overload of consults given in the primary level of medical attention.

Aggregation function utilized were (1) maximum function dichotomous variables, to obtain the value of 1 as the indicator of an action carried out during the study period, or the value of 0 if the action was not carried out during the four years; and (2) mean function for continuous variables, to reflect a single value throughout the study period. This aggregation strategy has been previously reported [24].

### 2.4. Data Analysis

Data are presented as the percentage of patients that met the goal for disease control and management. Control goals were categorized as “yes/no” for each patient, covering four years. The chi-squared test, odds ratio (OR), and 95% CI were employed to compare the different groups using the Crosstabs procedure. A *P* < 0.05 indicated a statistically significant difference. All statistical analyses were performed with SPSS version 20 software (IBM Corp., Armonk, NY, USA).

## 3. Results

The study population was made up of 45,498 patients, where a total of 41.01% had HBP, 30.09% had DM, and 28.90% had DM+HBP. Almost half of the consultations were given to the DM+HBP patients, despite them being the smallest group. The DM+HBP group had the highest amount of patients in pharmacological therapy (Table 2).

DM: diabetes mellitus; HBP: hypertension; SD: standard deviation; LDL: low-density lipoprotein; HDL: high-density lipoprotein.

Table 2 shows the percentages of patients that met established goals. The DM+HBP group had a lower amount of patients with glycemic control than the DM group, while they had higher hypertension control than the HBP group. Meanwhile, the DM+HBP group had almost 30% of their population in control for both.

Serum lipids got higher control in the DM+HBP group, while they also had higher control for the intersection of glycemic, hypertension, and LDL cholesterol. Also, this group had more population receiving clinical examinations and referrals to specialty consultation.

Table 3 compares the probability of reaching a goal between the groups. With simultaneous control of glycemia and hypertension, the DM+HBP group had 2.2-2.7-times less probability of achieving control than the DM and HBP groups. However, the DM+HBP group had more probability of reaching hypertension control than HBP, while also having less probability of reaching glycemic control than DM.

The DM+HBP patients had a 2-3-times greater probability of receiving pharmacologic therapy, nutritional education, serum lipids, clinical examination, and being referred to a specialist.

Patients in the DM group had a 20% less probability of achieving disease control than the patients in the HBP group while also having less probability of receiving pharmacologic therapy, nutritional guidance, and meeting the triglyceride goal. Also, the DM group patients had less probability of having foot and eye examinations than the HBP group patients.

Finally, the DM patients had more probability of being referred to an angiology, ophthalmology or internal medicine specialist and less probability of being referred to a cardiology specialist than the HBP patients. There were no differences in meeting the goal of cholesterol control between the patients in the DM group and those in the HBP group.

## 4. Discussion

Reaching a goal is the patient's shared responsibility through self-care and the physician through patient follow-up [13]. The coexistence of DM and HBP worsens clinical outcomes. Therefore, management should be comprised of a multifaceted approach [9].

A patient follow-up under standards, such as clinical foot and eye examinations and the periodic referral of patients to specialists, is an aspect that should be properly carried out before the implementation of other strategies.

The present study found that 45% of the diabetic and/or hypertensive patients reached control, but there were significant differences between the groups analyzed. In comparison, patients in DM+HBP reached control by 30%. Strikingly, the DM+HBP patients had better hypertension control (71%) but inferior glycemic control (43%).

A decrease in the occurrence and progression of retinopathy, nephropathy, and neuropathy has been demonstrated with glycemic control [25]. Within Latin America, glycemic control has been reported in 43% of patients, while in the United States, that proportion is 49% [26], and European studies reported 64% of patients in control [27]. In the present study, 48.6% of patients from the DM group reached glycemic control.

Regarding hypertension, the controlled disease was found in 56.8-58.7% of patients in other Mexican studies [3, 28]. Data collected in 2010 showed that the proportion of hypertension control was 50.4% in high-income countries, compared with 26.3% in low- and middle-income countries [11, 29]. In the present study, control is accomplished in 53% of the HBP group patients, similar to that reported for high-income countries.

Previous studies did not include patients with DM and HBP; this is a strength of the present study. We showed that there were differences between those groups of patients, at least in our study population.

The undiagnosed disease was 4% of HBP and 11% of DM, implying that at least 15% of the total population presented with both diseases and received treatment for only one of them.

Reported masked disease in 2000 was 29.3% for diabetic patients with masked HBP [9]. Having both diseases, but receiving treatment only for one, increases the damage to target organs and cardiovascular risk in those patients [9].

Serum lipid control is a less-studied theme. Only 3% of the patients had triglyceride control in our study population, and 5% had total cholesterol control. Moreover, control from LDL cholesterol and HDL cholesterol goals did not surpass 0.5% of the analyzed population.

Studies performed in the diabetic Mexican population reported LDL cholesterol control in 52% in 2003 and 12% in 2006 [30], while 57% had total cholesterol control [31]. In the United States, reports from LDL cholesterol control go from 40 to 63% in DM patients [26]. Variability in compliance with lipid control or other health goals even within Mexico may be due to substantial heterogeneity in healthcare quality assessments across Mexican healthcare subsystems. These differences across subsystems remained even after adjusting for socioeconomic, demographic, and health factors [31]. Despite regional variations, our population had the lowest control of serum lipids worldwide.

Nevertheless, our patients had glycemic control and hypertension control results similar to those reported for high-income countries; this could be due to an unequal therapeutic focus. More attention was given to controlling glycemia and hypertension than to serum lipid control.

These results suggest that therapeutic strategies for controlling lipid levels do not necessarily accompany glycemic control and hypertension control strategies. Healthcare quality was also measured through actions performed in the primary care setting, such as clinical examinations, along with a referral to specialists. Mexican studies showed that 8-25% of DM patients received an eye exam or were referred to an ophthalmologist, and 14-98% had a foot exam over one year [32].

Our analysis showed that only 1 out of every five patients had a foot or eye exam in the primary care setting within the study's 4-year time frame. During that same period, only 1% were referred to an angiologist or cardiologist, 5% to an ophthalmologist, and 14% to an internist. National and international guidelines indicate that a foot examination should be carried out at every consultation, and referrals should be once a year.

No previous reports evaluated healthcare quality in a population made up of patients with those three diagnoses. That division made it possible for us to analyze the differences between groups (see Table 3).

We found that the DM+HBP patients received more nutritional orientation and had a greater probability of being referred to than the single diagnosis groups. That could have influenced the greater control fulfillment with the hypertension goal but was incongruent with the inferior glycemic control observed in that group.

The DM+HBP patients had more than double the probability of having eye and foot examinations than the patients diagnosed only with DM or only with HBP. The DM+HBP patients had a 10-times greater probability of being referred to a cardiologist than the DM patients. The DM+HBP patients and the DM patients had a three- and 1.7-times greater probability than patients with HBP of being referred to an angiologist, respectively. In comparison, a more significant number of DM+HBP patients were referred to an angiologist than the DM patients. The DM+HBP patients were referred to an ophthalmologist 2.5 times more and to an internist 3 times more than the patients that were just diabetic or just hypertensive.

A direct comparison between the DM and HBP groups showed that the DM group also had less probability of having nutritional orientation, foot and eye examinations, and fewer cardiology referrals than the HBP group patients. In contrast, the DM group patients had a greater probability of being referred to angiology, internal medicine, and ophthalmology.

This data clarifies that the quality of chronic degenerative disease care within the same health system can vary, depending on the patients' diagnoses. We also observed that it was more common for a DM patient to have masked HBP than for an HBP patient to have masked DM.

We found low goal achievement in the primary care setting. Therefore, it is likely that complications are not detected opportunely and are diagnosed at advanced stages, reducing the patient's quality of life, increasing healthcare costs, and reducing patient life expectancy. It has been documented that diabetic patients have better control of their parameters when they are cared for by departments of specialized medicine in comparison with primary care physicians [33].

Strategies should be implemented in Mexico to improve this aspect of healthcare. It should be understood that the problem is not resolved by referring the patient to a specialist. The capacity of the specialized consultation must be calculated and the necessary adjustments made to meet the goals. Regarding the implemented strategies, simulating goal compliance by referring the patient to the specialist, even though the patient is never treated, or saturating the specialty consultation, to the detriment of quality care in both the programmed and emergency consultations, must be prevented.

Limitations for the present study included the time series analysis that was not possible to make given that time between subsequent consultations was not equally spaced for all the patients. For example, it was not possible to know the time during follow-up in which the therapy goals were achieved. The authors did not manipulate these records in any manner; therefore, a collapse of data had to be done to perform statistical analysis. Another important limitation was that there were no records of referrals by primary care physicians to the nephrology service. This was due to peculiarities of the evaluated health system, where the primary care physician refers to the internal medicine service, without being able to refer the patient directly to a nephrology consultation. Kidney complications are very important in both diabetic and hypertensive patients and more so if the patient has both conditions.

Mexico is a country in development, and given that the population for this study was taken from a public health care system, HbA1c is not measured as the guidelines state; therefore, authors agreed to take glycemic control according to preprandial and postprandial measures.

## 5. Conclusions

Glycemia and hypertension control was achieved in 50% of the population, similar to that in reports from developed countries. Incongruously, serum lipid control was lower than that reported worldwide, suggesting that the control of secondary variables (such as lipids) was not always conducted with the same quality as that of the primary aims.

Foot and eye examinations were performed between 12 and 22% of patients, varying according to their diagnoses, while referrals to a specialist, such as an angiologist or cradologist, can be in less than 1% of patients.. The DM+HBP patients had a higher number of general medicine and specialty consultations, better control of HBP and serum lipids, and inferior glycemic control. The amount of undiagnosed disease reached 15%.

The care given to patients treated through the health systems must improve. Feasible health education strategies should be developed, and their application designed to not interfere with the quality of the programmed consultations.

## Figures and Tables

**Table 1:** Control and management goals used in the present study.

Primary control points	Indicator
Systolic blood pressure	<140 mm Hg
Diastolic blood pressure	>65 and <90 mm Hg
Preprandial glycemia	<126 mg/dl
Postprandial glycemia	<200 mg/dl
Secondary control points	Indicator
Triglycerides	<150 mg/dl^∗^
Cholesterol	<200 mg/dl^∗^
LDL	<100 mg/dl^∗^
HDL	≥50 mg/dl^∗^
Management points	Indicator
Annual eye exam	Yes/no^∗∗^
Annual foot exam	Yes/no^∗∗^
Angiology referral	Yes/no^∗∗^
Cardiology referral	Yes/no^∗∗^
Internal medicine referral	Yes/no^∗∗^
Ophthalmology referral	Yes/no^∗∗^

^∗^Mean values during the study period. ^∗∗^Action performed at least 1 time during the study period (2012-2015). LDL: low-density lipoprotein; HDL: high-density lipoprotein.

**Table 2:** Percentage of patients reaching control and management goals.

Variables	Total *N* = 45498	DM+HBP *N* = 13692	DM *N* = 13147	HBP *N* = 18659
Per pathology	100%	28.90%	30.09%	41.01%
Consultations	100%	49.58%	16.82%	33.60%
Age (years ± SD)	56.6 ± 16.7	57.6 ± 18.3	51.6 ± 15.5	60.0 ± 14.4
Pharmacologic therapy	72.71%	86.75%	63.93%	68.59%
Glycemic controlled	66.25%	42.96%	48.60%	95.77%
Blood pressure controlled	69.03%	71.42%	88.99%	53.21%
Control of glycemia and blood pressure	44.78%	29.60%	48.60%	53.21%
Triglyceride goal	3.52%	5.40%	2.24%	3.03%
Cholesterol goal	5.27%	8.74%	3.92%	3.69%
LDL cholesterol goal	0.20%	0.44%	0.11%	0.10%
HDL cholesterol goal	0.13%	0.25%	0.09%	0.08%
Control of glycemia, blood pressure, and LDL cholesterol	0.04%	0.11%	0.03%	0.00%
Nutritional education	45.97%	60.76%	35.45%	42.53%
Clinical foot exam	22.29%	33.03%	16.36%	18.58%
Clinical eye exam	18.15%	27.18%	12.56%	15.47%
Angiology referral	0.84%	1.39%	0.80%	0.47%
Cardiology referral	1.63%	2.24%	0.22%	2.17%
Internal medicine referral	13.99%	23.31%	10.93%	9.30%
Ophthalmology referral	5.46%	9.50%	4.01%	3.51%

**Table 3:** Comparison between groups for reaching goals through odds ratio.

Variables	OR (95% CI) *P* value		
	DM+HBP vs. DM^∗^	DM+HBP vs. HBP^∗^	DM vs. HBP^∗^
Glycemic control	0.79 (0.75, 0.83) ≤ 0.001	—	—
Blood pressure control	—	2.19 (2.09, 2.30) ≤ 0.001	—
Glycemia and blood pressure control	0.44 (0.42, 0.47) ≤ 0.001	0.37 (0.35, 0.39) ≤ 0.001	0.83 (0.80, 0.87) ≤ 0.001
Pharmacologic therapy	3.69 (3.47, 3.92) ≤ 0.001	2.99 (2.82, 3.17) ≤ 0.001	0.81 (0.77, 0.85) ≤ 0.001
Nutritional guidance	2.82 (2.68, 2.96) ≤ 0.001	2.09 (2.00, 2.19) ≤ 0.001	0.74 (0.71, 0.78) ≤ 0.001
Triglyceride goal	2.49 (2.17, 2.85) ≤ 0.001	1.83 (1.64, 2.05) ≤ 0.001	0.74 (0.64, 0.85) ≤ 0.001
Cholesterol goal	2.34 (2.11, 2.61) ≤ 0.001	2.50 (2.27, 2.75) ≤ 0.001	1.07 (0.95, 1.20) 0.274
LDL goal	4.13 (2.31, 7.39) ≤ 0.001	4.56 (2.69, 7.72) ≤ 0.001	1.10 (0.55, 2.22) 0.781
HDL goal	2.72 (1.41, 5.26) 0.002	3.09 (1.68, 5.68) ≤ 0.001	1.14 (0.53, 2.43) 0.743
Foot exam	2.52 (2.38, 2.67) ≤ 0.001	2.16 (2.05, 2.27) ≤ 0.001	0.86 (0.81, 0.91) ≤ 0.001
Eye exam	2.60 (2.44, 2.77) ≤ 0.001	2.04 (1.93, 2.15) ≤ 0.001	0.78 (0.74, 0.84) ≤ 0.001
Angiology referral	1.75 (1.38, 2.22) ≤ 0.001	3.00 (2.33, 3.88) ≤ 0.001	1.72 (1.29, 2.29) ≤ 0.001
Cardiology referral	10.38 (7.08, 15.19) ≤ 0.001	1.04 (0.89, 1.20) 0.641	0.10 (0.07, 0.15) ≤ 0.001
Internal medicine referral	2.48 (2.32, 2.65) ≤ 0.001	2.97 (2.78, 3.16) ≤ 0.001	1.20 (1.11, 1.29) ≤ 0.001
Ophthalmology referral	2.51 (2.27, 2.79) ≤ 0.001	2.89 (2.62, 3.18) ≤ 0.001	1.15 (1.02, 1.29) 0.019

^∗^Reference group (OR = 1.0). With no comparison between groups because they did not share the evaluation goal. DM: diabetes mellitus; HBP: hypertension; LDL: low-density lipoprotein; HDL: high-density lipoprotein.

## Data Availability

The data belong to the Colima State Public Health Department and are only available to researchers that have applied for the data at the Administrative System of Clinical Records of Colima (SAECCOL, Spanish acronym), contact information: ivan_delgado_enciso@ucol.mx, contact person: Ivan Delgado.
